# Supplementary figures and images for: Fatty acid metabolism suppresses neonatal cardiomyocyte proliferation by increasing PDK4 and HMGCS2 expression through PPARδ
Source: PLoS One. 2025 May 8;20(5):e0318178. doi: 10.1371/journal.pone.0318178 (PMC12061097; doi:10.1371/journal.pone.0318178)

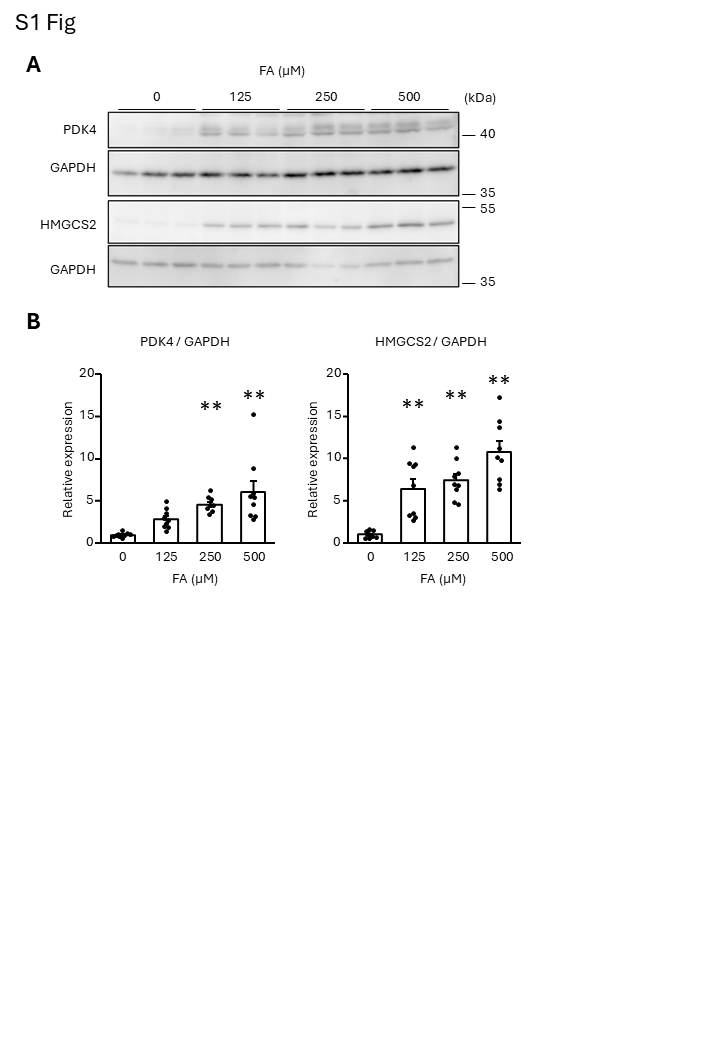

Supplement: S1 Fig — NRCMs were treated with 500 μM FA for 24 h. The protein expression of PDK4 and HMGCS2 was measured using western blotting with anti-PDK4 and anti-HMGCS2 antibodies. (A) Representative images. (B) Quantitative data. Data are shown as mean ± SEM (n = 6). **p < 0.01 by Student’s t-test. (TIF) [file pone.0318178.s001.tif]

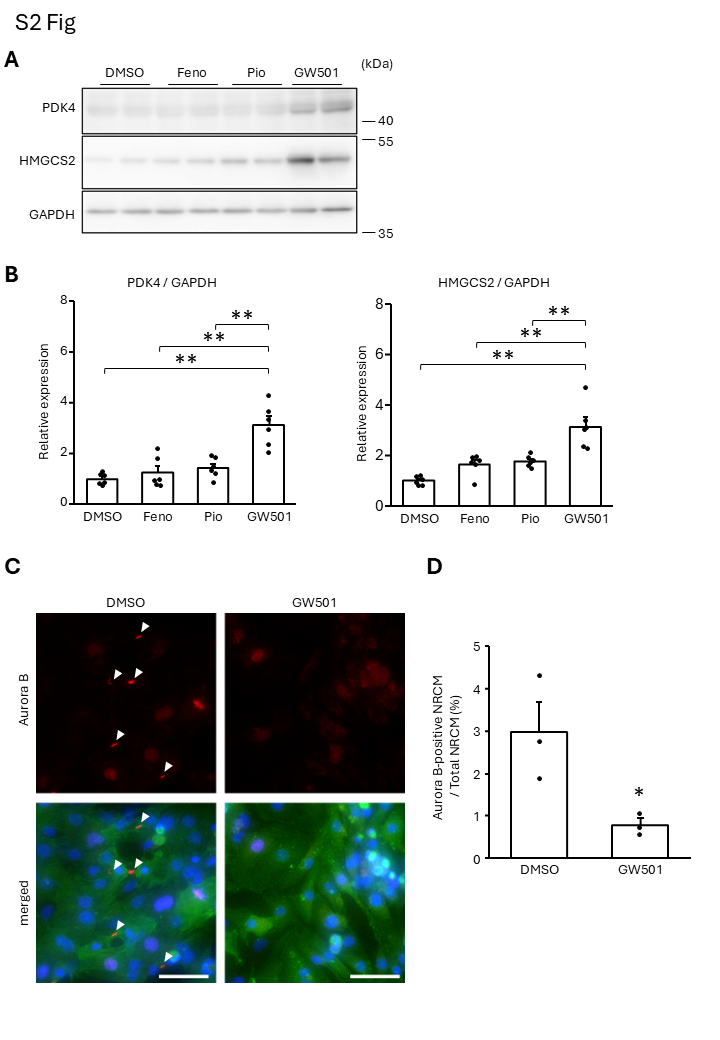

Supplement: S2 Fig — NRCMs were treated with fenofibrate (Feno), pioglitazone (Pio), or GW501516 (GW501) at 10 μM for 24 h. (A, B) Protein expression was measured using western blotting with anti-PDK4 and anti-HMGCS2 antibodies. (C, D) The proportion of Aurora B-positive NRCMs was analyzed by immunostaining. Cells were stained with an anti-Aurora B antibody (red). Cardiomyocytes and nuclei were labeled with anti-α-actinin antibody (green) and DAPI (blue), respectively. (A, C) Representative images. (B, D) Quantitative data. The bars indicate 100 μm. Arrowheads indicate Aurora B. Data are shown as mean ± SEM (A: n = 6, B: n = 3). *p < 0.05, ** p < 0.01 by Dunnett test (A) and Student’s t-test (B). (TIF) [file pone.0318178.s002.tif]

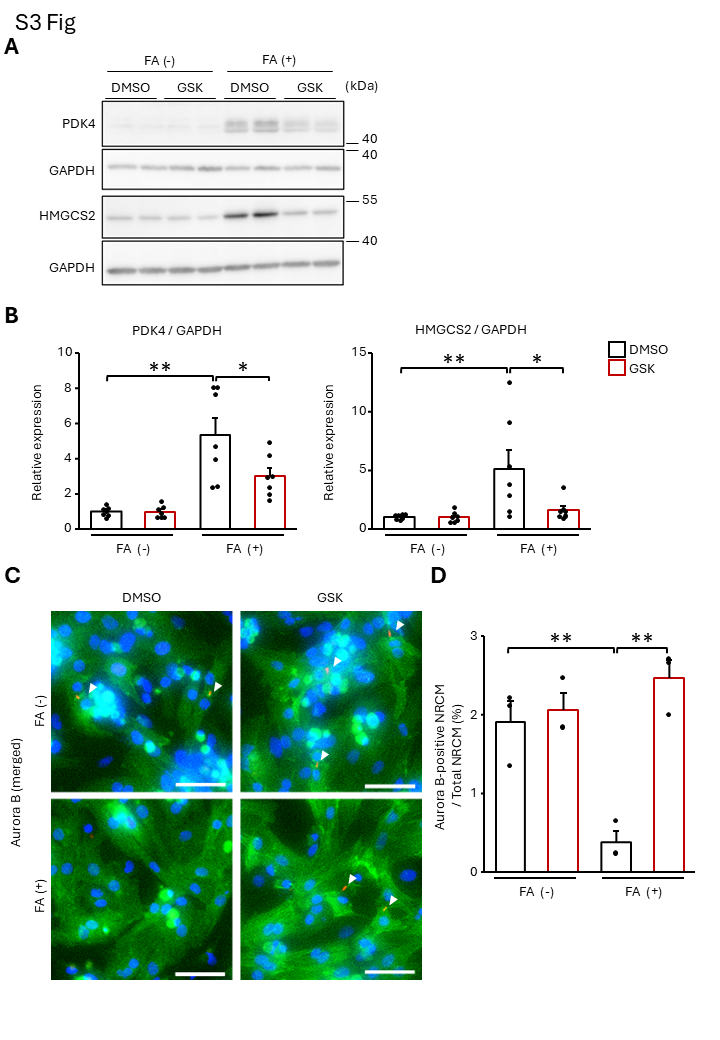

Supplement: S3 Fig — NRCMs were pretreated with GSK3787 (GSK) at 10 μM for 24 h, followed by the treatment of 500 μM FA for 24 h. (A, B) Protein expression was measured using western blotting with anti-PDK4 and anti-HMGCS2 antibodies. (C, D) The proportion of Aurora B-positive NRCMs was analyzed by immunostaining. Cells were stained with an anti-Aurora B antibody (red). Cardiomyocytes and nuclei were labeled with anti-α-actinin antibody (green) and DAPI (blue), respectively. (A, C) Representative images. (B, D) Quantitative data. The bars indicate 100 μm. Arrowheads indicate Aurora B. Results are shown as mean ± SEM (A: n = 7, B: n = 3). *p < 0.05, **p < 0.01 by Tukey-Kramer test. (TIF) [file pone.0318178.s003.tif]

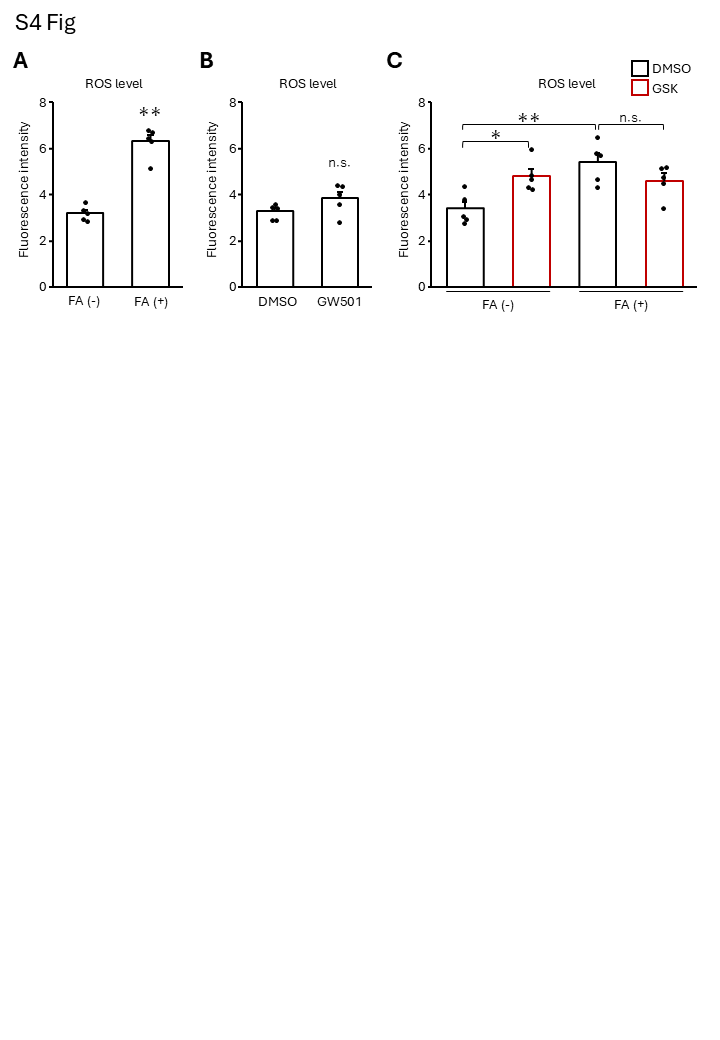

Supplement: S4 Fig — (A, B) Reactive oxygen species (ROS) levels were measured using the fluorescence of CellROX Green 24 h after the stimulation with 500 μM FA (A) or 10 μM GW501516 (GW501) (B) in NRCMs. Data are shown as mean ± SEM (n = 5). **p < 0.01 by Student’s t-test. (C) Data are shown as mean ± SEM (n = 5). *p < 0.05, **p < 0.01 by Tukey-Kramer test. n.s. indicates no significance. (TIF) [file pone.0318178.s004.tif]

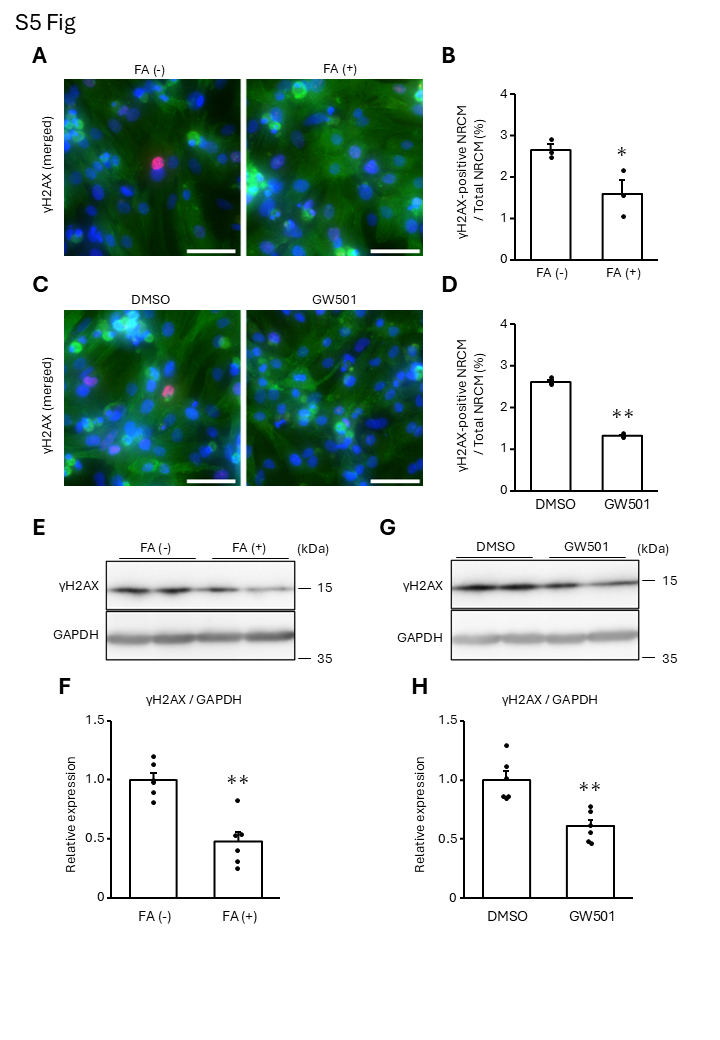

Supplement: S5 Fig — NRCMs were treated with 500 μM FA (A, B, E, F) or 10 μM GW501516 (GW501) (C, D, G, H) for 24 h. (A-D) The proportion of γH2AX-positive NRCMs was analyzed by immunostaining. Cells were stained with an anti-γH2AX antibody (red). Cardiomyocytes and nuclei were labeled with anti-α-actinin antibody (green) and DAPI (blue), respectively. The bars indicate 100 μm. (E-H) Protein expression was measured by western blotting with anti-γH2AX antibody. (A, C, E, G) Representative images. (B, D, F, H) Quantitative data. Data are shown as mean ± SEM (A, B: n = 3, C, D: n = 6). *p < 0.05, **p < 0.01 by Student’s t-test. (TIF) [file pone.0318178.s005.tif]

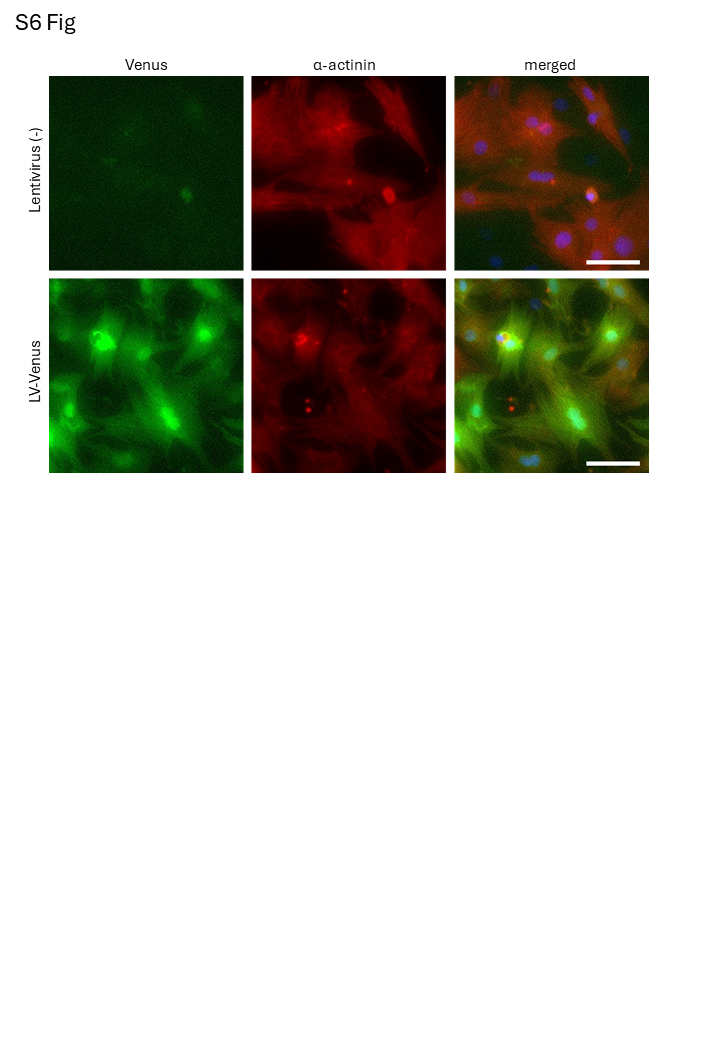

Supplement: S6 Fig — The NRCMs were infected with the indicated lentiviral vectors for 72 h. (A) Transfection efficacy was estimated using Venus expression (green). Cardiomyocytes and nuclei were labeled with anti-α-actinin antibody (red) and DAPI (blue), respectively. Representative images are shown. The bars indicate 100 μm. (TIF) [file pone.0318178.s006.tif]

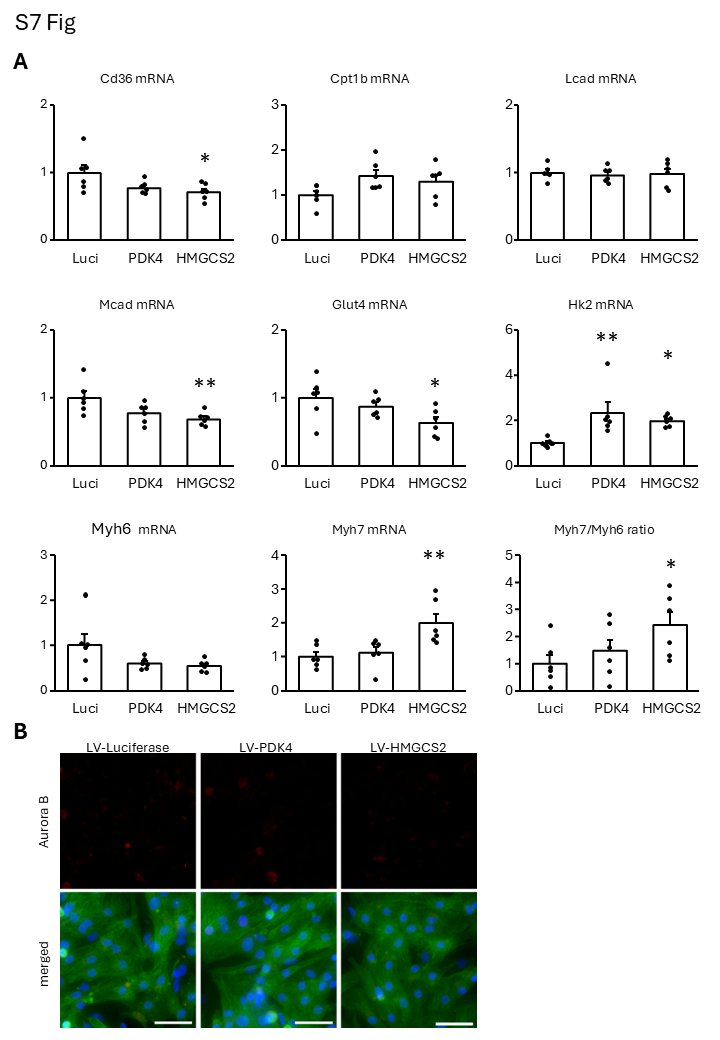

Supplement: S7 Fig — (A) NRCMs were infected with the indicated lentiviral vectors for 48 h, followed by the treatment of 500 μM FA for 24 h. Transcript expression was measured using real-time RT-PCR. (B) The proportion of Aurora B-positive NRCMs was analyzed by immunostaining. Cells were stained with anti-Aurora B antibody (red). Cardiomyocytes and nuclei were labeled with anti-α-actinin antibody (green) and DAPI (blue), respectively. The bars indicate 100 μm. Results are shown as the mean ± SEM (n = 6). **p < 0.01 vs. LV-Luciferase by Dunnet test. (TIF) [file pone.0318178.s007.tif]

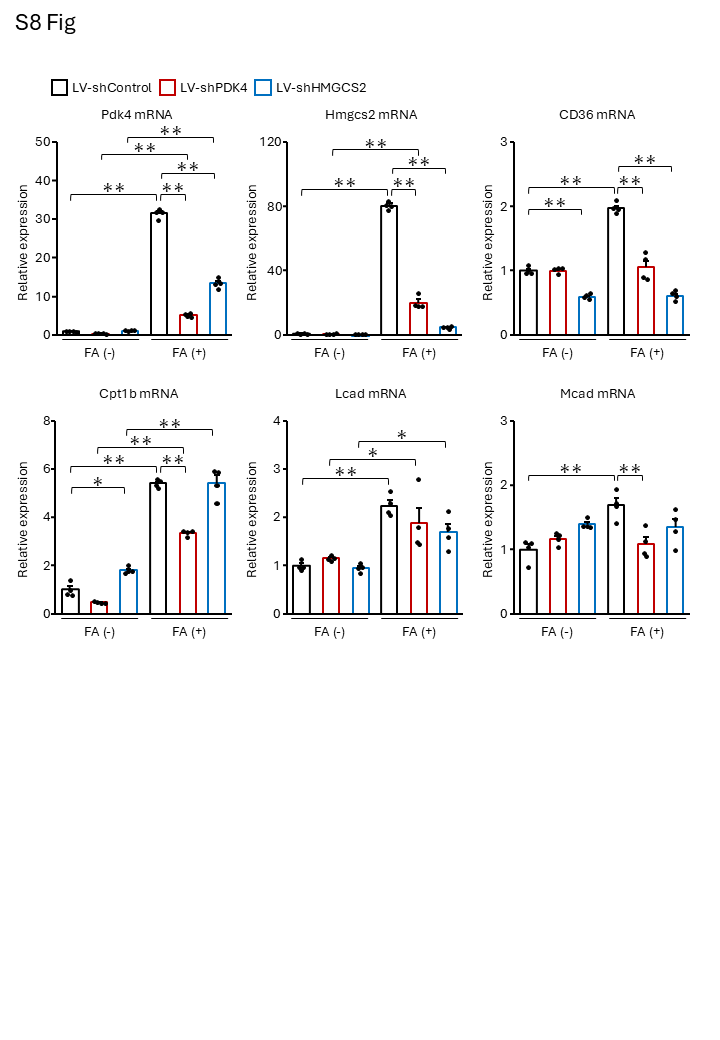

Supplement: S8 Fig — The NRCMs were infected with the indicated lentiviral vectors for 48 h, followed by the stimulation of 500 μM FA. The expression of transcripts was measured by real-time RT-PCR. Results are shown as mean ± SEM (n = 4). *p < 0.05, **p < 0.01 by Tukey-Kramer test. (TIF) [file pone.0318178.s008.tif]

Figure 6A

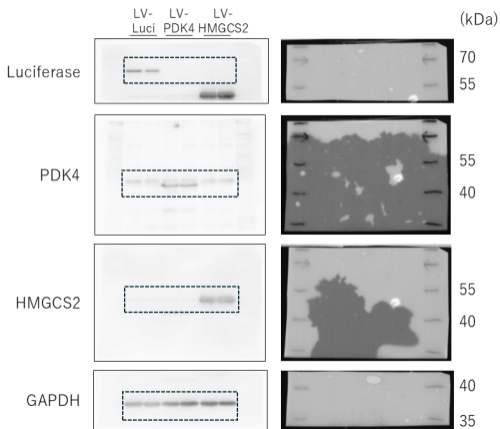

Figure 7A

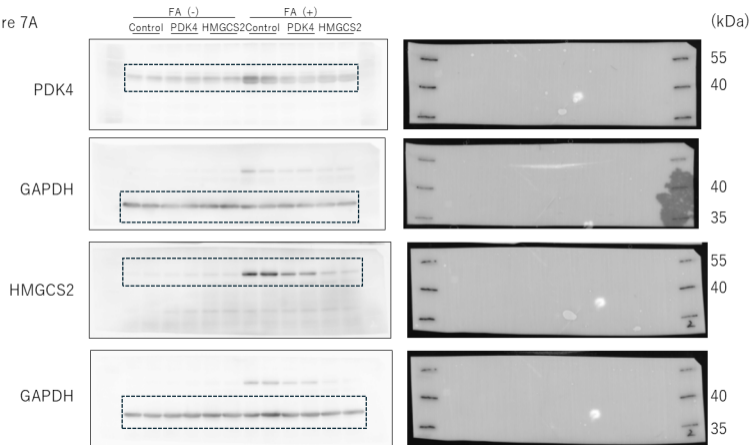

S1 Fig

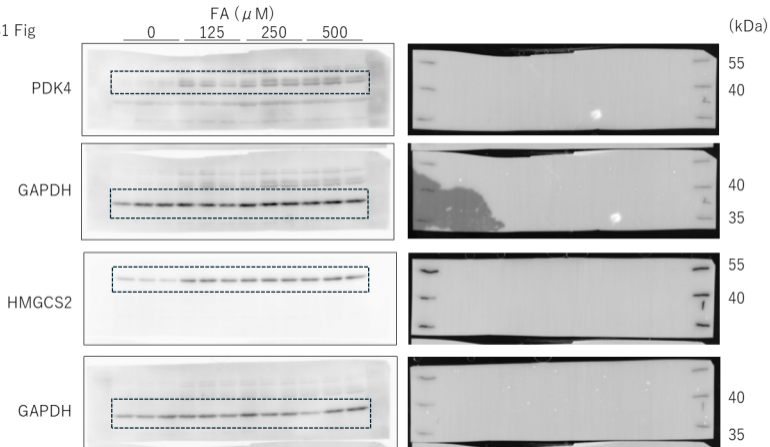

S2 Fig

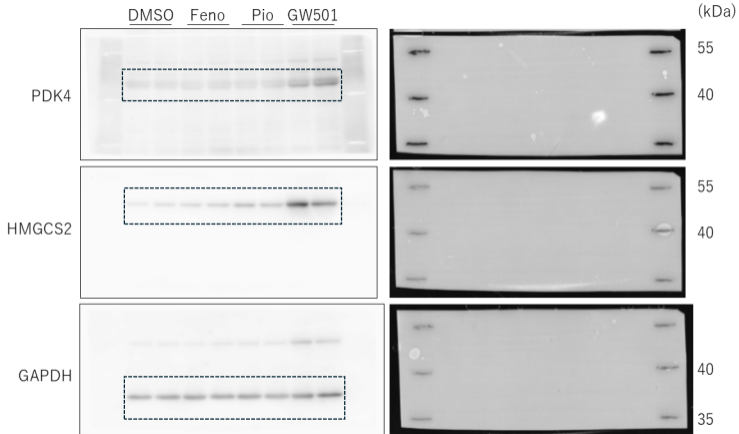

S3 Fig

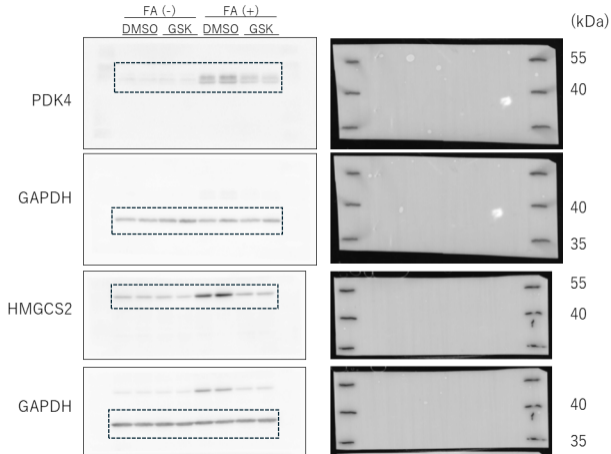

S5 Fig

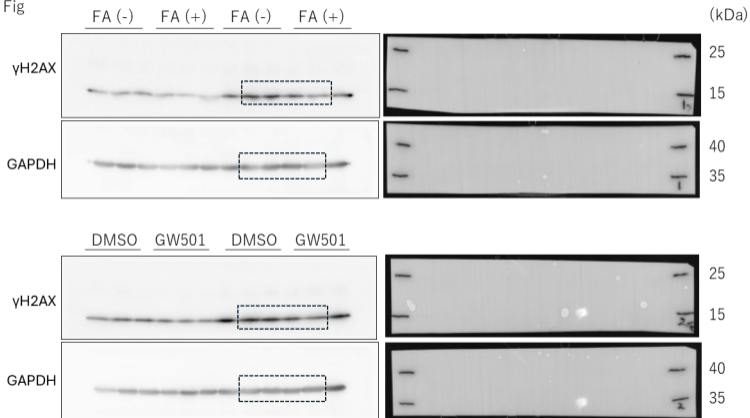

Supplement: S1 raw images — (PDF) [file pone.0318178.s009.pdf]
