# Supplementary material for: Fatty acid metabolism suppresses neonatal cardiomyocyte proliferation by increasing PDK4 and HMGCS2 expression through PPARδ
Source: PLoS One. 2025 May 8;20(5):e0318178. doi: 10.1371/journal.pone.0318178 (PMC12061097; doi:10.1371/journal.pone.0318178)
Supplement: Attachment — Submitted filename: Response to reviewers.docx [file pone.0318178.s011.docx]

The Editor

PLOS ONE

Manuscript number: PONE-D-24-32166

Please find the revised manuscript entitled “Fatty acid metabolism suppresses neonatal cardiomyocyte proliferation by increasing PDK4 and HMGCS2 expression through PPARδ“ by Tanaka, S. et al. We revised the manuscript according to the reviewers’ comments. The responses to the comments are described as “comments for revision”. We thank the editors and reviewers for their comments, which we believe have improved our manuscript. We hope that this manuscript will be reconsidered for publication in PLOS ONE.

All authors have read this manuscript and agree to submit it to your journal. We haven’t submitted any results of this manuscript elsewhere.

This study is partially supported by MEXT/JSPS KAKENHI Grants 20K22707 to ST, 22K15277 to ST. This research was also supported by Basis for Supporting Innovative Drug Discovery and Life Science Research (BINDS) from AMED under grant numbers JP23ama121052 and JP23ama121054. There was no additional external funding received for this study. In addition, the authors have no conflicts of interest associated with this manuscript.

~ Comments for Revision ~

Reviewer #1

**Comment #1:** Fig. 1 and 3: The authors should show the protein upregulations of PDK4 and HMGCS2 in neonatal cardiomyocytes stimulated with FA.

**Response:** According to the reviewer’s comment, we measured PDK4 and HMGCS2 protein expressions and confirmed FA and GW501516 increased both expressions. These results are shown in S1 Fig and S2 Fig.

**Comment #2:** Fig. 1: The authors described that excess acetyl-CoA is likely to be consumed for the metabolic processes other than ATP production, such as ketogenesis. Did the authors assess acetyl-CoA levels in cardiomyocytes with FA? Also, ketone and/or the metabolites are increased in the myocytes stimulated with FA?

**Response:** According to the reviewer’s comment, we measured acetyl-CoA and β-hydroxybutyrate, a ketone body, in FA-treated cardiomyocytes. Although FA upregulated acetyl-CoA and Hmgcs2 level, β-hydroxybutyrate level was not altered by FA treatment. These data indicate the addition of FA upregulated several enzymes of β-oxidation and a ketogenic factor without ATP or ketone body production. These results are shown in Figure 1B and 1E.

**Comment #3:** Fig. 2: The readers would wonder if neonatal cardiomyocytes are actually proliferating at basal condition. Ki67 and pHH3 are used for cell-cycling indicators. But it is still unclear if cytokinesis and karyokinesis are happening in the cardiomyocytes since mouse cardiomyocyte is an endomitotic cell. To clarify this concern, it would better to assess expression levels of Aurora kinase B, Anillin, and Prc1 at transcript and protein levels. Immunocytochemistry also can detect the expressions of these proteins. Furthermore, the number of cells should be assessed to see the proliferation.

**Response:** We thank the reviewer for the important suggestion. As suggested by the reviewer, Aurora B, Anillin, and Prc1 mRNA were measured using real-time PCR and little alteration was observed by FA. Therefore, we analyzed NRCMs cytokinesis using immunostaining with an anti-Aurora B antibody and revealed that FA reduced intracellular Aurora B. Moreover, we quantified the number of NRCMs using the WST-8 assay and confirmed that there were fewer NRCMs in FA contained condition. These data indicate that cytokinesis ocurrs in NRCMs during proliferative process. Moreover, FA suppresses proliferation in NRCMs. These results are shown in Figure 2E, 2F, and 2G.

**Comment 4:** Fig. 2: What is the consequence in Ki67 negative neonatal cardiomyocytes stimulated with FA? Do they maturate quickly? Evaluation of binucleation and sarcomere component isoform switch will answer this question.

**Response:** As suggested by the reviewer, we measured the transcript expression of sarcomere components, such as Myh6 and Myh7, and revealed that FA suppressed Myh7 mRNA without the change of Myh6. In addition, the ratio of Myh7/Myh6 was also reduced after FA treatment, suggesting FA promotes NRCM maturation. The results were shown in Figure 5. We tried the experiment to evaluate NRCMs binucleation. However, we failed to analyze binucleation due to technical limitation: To check the number of nuclei, we seeded cells sparsely than usual, resulting in the severe damage of NRCMs in the presence of FA.

**Comment #5:** Fig. 3 and 4: Again many readers will have a question about proliferation and maturation in neonatal cardiomyocytes with PPARdelta agonist and antagonist.

**Response:** According to the reviewer’s comment, we measured Aurora B expression and Myh7 mRNA in NRCMs treated by PPARδ agonist- or antagonist to evaluate proliferation and maturation, respectively. A PPARδ agonist GW501516 reduced Aurora B-positive NRCMs, accompanied by the decreases expression of Myh7 mRNA. Moreover, a PPARδ inhibitor GSK3787 regained the reduction of Aurora B-positive NRCMs, accompanied by the increase in Myh7 mRNA. These results indicate that PPARδ activation promotes NRCM maturation. The results were shown in Figure 5, S2 Fig, and S3 Fig.

**Comment #6:** Fig. 5B and C: If cardiomyocytes are not stimulated with FA in Fig. 5B, can ROS be increased without beta-oxidation substrates? Furthermore, since there is a difference in ROS levels of cardiomyocytes stimulated with FA alone between Fig. 5A and 5C, it is still inconclusive about that GSK did not suppress FA-induced ROS production in Fig. 5C. How can the readers interpret this inconsistency?

**Response:** We apologize for the confusing presentation. In Fig. 5B (original), we examined whether a PPARδ agonist GW501516 increased ROS level without FA stimulation. The treatment with GW501516 only, without β-oxidation substrates, did not increase ROS. Furthermore, when the absolute value of fluorescence intensity was compared, there was no significant difference in the FA alone group between Fig. 5A and 5C (data not shown). In the experiments, we examined whether ROS-induced DNA damage was involved in NRCMs cell cycle arrest. Although FA increased ROS level in NRCMs, FA or GW501516 did not increase γH2AX, a damaged DNA marker. These data suggest that FA or GW501516 did not stop NRCMs proliferation through ROS-induced DNA damage. Since the effects of a PPARδ inhibitor GSK3787 on ROS level in NRCMs was obscure in Fig 5C (original), we consider that the results of ROS analysis were suitable for supportive data and original Figure 5 was transferred to S4 Fig. The results of γH2AX expression were shown in S5 Fig and the discussion was described at Line 512.

**Comment #7:** Can shRNA-PDK4 and/or shRNA-HMGCS2 rescue the cell cycle arrest by FA stimulation or PPARdelta agonist?

**Response:** As suggested by the reviewer, we suppressed PDK4 and HMGCS2 using shRNA expressing lentiviral vectors and measured Ki67-positive NRCMs after FA stimulation. Silencing either PDK4 or HMGCS2 recovered FA-induced NRCMs cell cycle arrest. The results were shown in Figure 7.

**Comment #8:** Fig. 6: Did overexpressions of PDK4 and HMGCS2 change proliferation and maturation of neonatal cardiomyocytes after cell-cycling arrest?

**Response:** We apologize to the reviewer for our lack of understanding. Overexpressing PDK4 or HMGCS2 might still stop cell cycle after cell cycle arrest. In addition, we analyzed proliferation and maturation in PDK4- or HMGCS2-overexpressing NRCMs. We revealed that Aurora B was not detected in Luciferase-overexpressing control NRCMs as well as PDK4- or HMGCS2-overexpressing cells after cell-cycling arrest. Moreover, PDK4 overexpression did not alter Myh7 mRNA and HMGCS2 overexpression rather increased Myh7. The results were shown in S7 Fig and possible reasons were discussed at Line 496.

Minor
**Comment #1:** Since neonatal cardiomyocytes and adult cardiomyocytes are completely different, it is recommended to use the word “neonatal cardiomyocytes” in the title.

**Response:** According to the reviewer’s comment, we change the title to “Fatty acid metabolism suppresses neonatal cardiomyocyte proliferation by increasing PDK4 and HMGCS2 expression through PPARδ“.

**Comment #2:** Given that there are multiple controls, it is better to use one-way ANOVA with post hoc test in Fig. 4.

**Response:** We thank the reviewer for the important suggestion. Based on your suggestion, we used one-way ANOVA followed by Tukey-Kramer test in Figure 4.

Reviewer #2:

Major issues

**Comment #1:** Line 123 “fatty acid mixture (FA) containing palmitic acid, oleic acid, and L-carnitine at a 1:1:2 molar ratio”: What is this mixture trying to model? Please clarify the situation in human body that is mimicked by this mixture for cardiomyocytes beyond the abundance of each FA.

**Response:** We thank the reviewer for the important suggestion. We used FA to mimic neonatal heart condition to be fed mother milk. Thus, we described in manuscript as follow:

To investigate the effects of fatty acids on NRCMs in neonatal model, we used an FA, which was the mixture of palmitic acid and oleic acid. Palmitic acid is one of the most abundant saturated fatty acids in mother milk. Oleic acid is also contained in mother milk plentifully and attenuates lipotoxicity induced by palmitic acid (21, 22).

**Comment #2:** Line 247 “Besides, excess acetyl-CoA is likely to be consumed for the metabolic processes other than ATP production, such as ketogenesis.”: The logic here is obscure. Please clarify why the authors speculated as stated.

**Response:** We apologize to the reviewer for our lack of reason. We confirmed that FA increased acetyl-CoA. The result was shown in Figure 1B.

**Comment #3:** The inhibition of PDK4 and HMGCS2 is obscure whether to inhibit PPARδ -> β oxidation pathway or not. Please consider rescue experiments to show this.

**Response:** According to the reviewer’s comment, we measured the transcript expressions of β-oxidation enzymes in either PDK4 or HMGCS2 silencing NRCMs. Suppressing PDK4 reduced FA-induced β-oxidation enzymes, including Cd36, Cpt1b, and Mcad, whereas suppressing HMGCS2 did not any except for Cd36 mRNA. These results indicate that the suppression of PDK4 inhibits PPARδ/β-oxidation pathway, while not that of HMGCS2.

**Comment #4:** Line 366: The FA stimulation is not directly shown to activate PPARδ in this manuscript. Please indicate if this has been shown previously.

**Response:** We apologize to the reviewer for our misleading words. I completely agree with you. Although some reports demonstrated that PPARδ was essential to promote fatty acid oxidation (Cheng, 2004; Palomer, 2016), there were few attestations to show free fatty acids directly activated PPARδ. In our study, FA did not increase the transcript expression of PPARδ (data not shown). Thus, we rewritten manuscript to as follow;

In the present study, we demonstrated that free fatty acids suppressed cardiomyocyte proliferation through the activation of PPARδ, …

**Comment #5:** If there is publicly available screening data using PPARs for cardiomyocytes, it might be interesting to see the difference between them to make the observed differences in this study.

**Response:** We thank the reviewer for the valuable comment. As described in Line 504, we discussed this point considering the RNA-seq data using iPS-CMs treated with a PPARδ agonist, reported by Wickramasinghe et al.
